# Current dialyzer classification in Japan and mortality risk in patients undergoing hemodialysis

**DOI:** 10.1038/s41598-024-60831-y

**Published:** 2024-05-04

**Authors:** Masanori Abe, Kan Kikuchi, Atsushi Wada, Shigeru Nakai, Eiichiro Kanda, Norio Hanafusa

**Affiliations:** 1 Committee of Renal Data Registry, Japanese Society for Dialysis Therapy, Tokyo, Japan; 2 Division of Nephrology, Hypertension, and Endocrinology, Department of Medicine, Nihon University School of Medicine, Tokyo, Japan; 3 Division of Nephrology, Shimoochiai Clinic, Tokyo, Japan; 4 Department of Nephrology, Kitasaito Hospital, Asahikawa, Japan; 5 Department of Clinical Engineering, Fujita Health University, Aichi, Japan; 6 Department of Medical Science, Kawasaki Medical School, Kurashiki, Japan; 7 Department of Blood Purification, Tokyo Women’s Medical University, Tokyo, Japan

**Keywords:** Albumin sieving coefficient, β_2_-microglobulin, Hemodialysis, Mortality, Super high-flux dialyzer, Nephrology, Renal replacement therapy

## Abstract

Dialyzers are classified into five types based on their β_2_-microglobulin clearance rate and albumin sieving coefficient: Ia, Ib, IIa, and IIb. In addition, a new classification system introduced a type S dialyzer. However, limited information is available regarding the impact of dialyzer type on patient outcomes. A cohort study was conducted using data from the Japanese Society for Dialysis Therapy Renal Data Registry database. Total 181,804 patients on hemodialysis (HD) were included in the study, categorized into four groups (type Ia, IIa, IIb, and S). The associations between each group and two-year all-cause mortality were assessed using Cox proportional hazard models. Furthermore, propensity score-matching analysis was performed. By the end of 2019, 34,185 patients on dialysis had died. After adjusting for all confounders, the risk for all-cause mortality was significantly lower in the type IIa, and S groups than in the type Ia group. These significant findings were consistent after propensity score matching. In conclusion, our findings suggest that super high-flux dialyzers, with a β_2_-microglobulin clearance of ≥ 70 mL/min, may be beneficial for patients on HD, regardless of their albumin sieving coefficient. In addition, type S dialyzers may be beneficial for elderly and malnourished patients on dialysis.

**Trial registration number:** UMIN000018641

## Introduction

Dialyzers are commonly classified as low-flux or high-flux membrane dialyzers. Low-flux membrane dialyzers are characterized by an ultrafiltration rate < 15 mL/mmHg/h and a β_2_-microglobulin (β2MG) clearance rate < 15 mL/min^1^. They effectively remove small solutes through diffusion, but only minimal amounts of middle-sized solutes, which are considered more toxic and more difficult to remove by diffusion^2^. This limitation led to the development of high-flux membrane dialyzers, which are defined by an ultrafiltration rate ≥ 15 mL/mmHg/h and a β2MG clearance rate ≥ 15 mL/min^1^. High-flux membranes have high hydraulic permeability and greater solute permeability for middle-sized solutes compared to low-flux membrane dialyzers. In 2005, to remove an expanded range of larger middle-molecular-weight molecules, super high-flux membranes with large pore sizes were developed in Japan^3^. In Japan, dialyzers were categorized into five types based on β2MG clearance: types I, II, III, IV, and V, with β2MG clearance rates of < 10, ≥ 10–30, ≥ 30–50, ≥ 50–70, and ≥ 70 mL/min, respectively, at a blood flow rate of 200 mL/min and a dialysate flow rate of 500 mL/min from 2005 to 2012^4,5^. By 2008, > 90% of Japanese patients were receiving hemodialysis (HD) with type IV or V dialyzers^6,7^.

In 2013, the dialyzer classification in Japan underwent revision^7^. Initially, dialyzers were categorized into two types based on β2MG clearance rates of 70 mL/min. Type I and II dialyzers were defined as having β2MG clearances lower or higher than 70 mL/min respectively. Furthermore, type I and II dialyzers were further divided into nonprotein permeable or low-permeable types (type a) and protein-permeable types (type b), with an albumin sieving coefficient (SC) of 0.03 serving as the reference value. Consequently, dialyzers were categorized into four types: Ia, Ib, IIa, and IIb, based on the combination of β2MG clearance and albumin SC. In addition, a new classification system introduced a type S dialyzer. Type S dialyzers were defined as having higher biocompatibility, enhanced solute removal through adsorption, and anti-inflammatory and antioxidant properties, which were difficult to evaluate using conventional solute removal measures such as urea and β2MG clearance. Therefore, dialyzers are currently classified into five types in Japan: Ia, Ib, IIa, IIb, and S.

HD using types IV and V dialyzers has been reported to reduce mortality rates compared with HD using types I, II, or III dialyzers. Additionally, type V dialyzers have been reported to be superior to type IV dialyzers in the old dialyzer classification^8,9^. However, there is limited information available on which type of dialyzer in the current classification leads to favorable outcomes. To address this gap, this study used data from a large-scale registry of dialysis patients in Japan to investigate the impact of dialyzers on clinical outcomes in patients undergoing HD, based on the current Japanese dialyzer classification.

## Methods

### Study design

This is a prospective cohort study that used data from the Japanese Society for Dialysis Therapy (JSDT) Renal Data Registry (JRDR) system, a nationwide cohort of patients on dialysis in Japan. Detailed information about the JRDR has been previously published^10,11^. The JSDT conducts an annual survey of all dialysis units in Japan, with response rates consistently exceeding 95% throughout the study period. The study protocol was approved by the Medicine Ethics Committee of JSDT (Approval No. 53), and the study was conducted in accordance with the principles outlined in the Declaration of Helsinki. The Ethics Committee waived the need for consent to use the JRDR data. The database has been fully de-identified to protect the privacy of the individuals involved, and any secondary or unauthorized use (i.e., any distribution to a third party, unauthorized replication or manipulation of the database, or deviation from the proposal accepted by the Committee of Renal Data Registry) has been strictly prohibited under the agreement between the principal investigators and JSDT, which retains all rights to the database. This study was registered at the University Hospital Medical Information Network (UMIN000018641).

### Setting and participants

Among patients undergoing maintenance HD at the end of 2017, with the observation period lasting until the end of 2019, those who underwent maintenance HD three times a week and had received maintenance dialysis for at least six months by the end of 2017 were included. However, patients were excluded if they were dialyzed less than three times a week or for less than three hours per session, had received hemodiafiltration (HDF) or peritoneal dialysis, had a history of organ transplantation, were under 18 years old, or had missing data on date of birth, dialysis initiation, type of dialyzer, or outcomes. Additionally, patients treated with type Ib dialyzers were excluded due to their negligible number. The main outcome measure for this study was the time to all-cause mortality during the two-year observation period. Patients were categorized into four groups based on the Japanese dialyzer classification, which was determined by β2MG clearance and albumin SC at baseline.

### Definition of the dialyzer type

Since 2013, dialyzer types in Japan have been classified based on β2MG clearance and albumin SC^7^. Type Ia dialyzers have β2MG clearance rates of < 70 mL/min and albumin SC < 0.03. Type Ib dialyzers have β2MG clearance rates of less than 70 mL/min and albumin SC ≥ 0.03. Type IIa dialyzers have β2MG clearance rates of ≥ 70 mL/min and albumin SC < 0.03. Type IIb dialyzers have β2MG clearance rates of ≥ 70 mL/min and albumin SC ≥ 0.03. Type S dialyzers possess special functions such as higher biocompatibility, solute removal by adsorption, and anti-inflammatory and antioxidant properties. Type S dialyzers represent a distinct class of dialyzers, different from conventional ones that are based on urea and β2MG clearances. Types Ia and IIa are characterized as protein non- or low-permeable dialyzers, while types Ib and IIb are characterized as protein-permeable dialyzers based on albumin SC. To measure urea and β2MG clearance and albumin SC, the performance evaluation in the bovine blood system is repeated at least three times under the conditions specified by the JSDT. The average value is used to determine the dialyzer classification. Supplementary Fig. S1, Supplementary Tables S1, S2, and S3 depict a more detailed information on the old and current dialyzer classifications in Japan and the dialyzers used in this study.

### Statistical methods

The data in this study were summarized using appropriate descriptive statistics, including proportions, means with standard deviations, percentages, or medians with interquartile ranges. Categorical variables were analyzed using the chi-squared test, while continuous variables were compared using the Student’s *t*-test, as appropriate. For comparing categorical data between groups, repeated-measures analysis of variance with Tukey’s honestly significant difference test or the Kruskal–Wallis test was used, as appropriate.

Baseline patient and laboratory data were collected from the JRDR database in 2017. These variables included age, gender, dialysis duration, modality, body mass index (BMI) at post-HD, cause of end-stage kidney disease, systolic and diastolic blood pressures (BPs), single-pool Kt/V, and laboratory measures including pre-HD hemoglobin, serum albumin, phosphate, calcium, intact parathyroid hormone (i-PTH), β2MG, and C-reactive protein (CRP) levels. Additionally, the history of myocardial infarction, cerebral hemorrhage, cerebral infarction, and limb amputation was also recorded.

The survival of patients according to dialyzer type was estimated using the Kaplan–Meier method and compared using the log-rank test. To assess whether baseline basic factors such as age, gender, cause of end-stage kidney disease, and dialysis duration predicted survival during the two-year follow-up period, Cox proportional hazards regression was performed. Additional analyses were conducted after adjusting for dialysis-related factors, including Kt/V, β2MG levels, and systolic and diastolic BPs. Furthermore, analyses were performed with adjustments for nutrition- and inflammation-related factors, including BMI, serum albumin, hemoglobin, phosphate, calcium, i-PTH, and CRP levels. In these analyses, age, β2MG levels, CRP levels, and hemoglobin levels were treated as continuous variables. Finally, the associations between all-cause mortality and the four dialyzer types based on β2MG clearance and albumin CS were examined.

Propensity score matching (PSM) was used to adjust for significant baseline covariates. The propensity scores were calculated using the aforementioned basic factors, dialysis-related factors, and nutrition- and inflammation-related factors. These propensity scores were then used in a univariate Cox proportional hazards regression analysis. Specifically, patients with type Ia dialyzers (used as the reference group) were matched in a 1:1 ratio with patients using other types of dialyzers. Then, patients receiving HD with type IIa dialyzer (the reference group) were matched with those receiving HD with type IIb dialyzer at a 1:1 ratio. In the PSM analysis, the propensity scores were derived from variables such as age, gender, dialysis vintage, comorbid cardiovascular disease (CVD) and diabetes mellitus (DM), systolic and diastolic BPs, BMI, Kt/V, β2MG, serum albumin, hemoglobin, phosphate, calcium, i-PTH, and CRP levels. The all-cause mortality was compared among the propensity score-matched patients.

When appropriate, missing covariate data were imputed using a conventional method for multivariate regression. All analyses were performed using JMP® version 13.0 (SAS Institute, Cary, NC, USA). The significance level was set at a *p*-value < 0.05.

## Results

### Baseline characteristics of the patients

**Figure 1:**
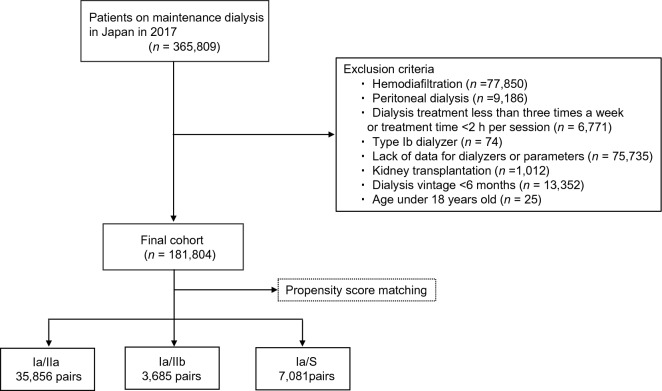
Flow diagram illustrating the process of patient selection.

At the end of 2017, a total of 365,809 patients were initially enrolled in the study. After applying the exclusion criteria, 181,804 patients remained for analysis (Fig. 1). The baseline characteristics of the patients in the four groups are summarized in Table 1. In the Ia and S groups, there were more elderly and female patients, a shorter dialysis vintage, higher rates of comorbid CVD, a lower BMI, lower serum albumin levels, and lower Kt/V values. In the Ia group, the distributions of types I–IV dialyzers, which are classified as old dialyzers in Japan, were 0.8%, 1.3%, 9.2%, and 88.7%, respectively. During the two-year observation period from January 2018 to December 2019, a total of 34,185 patients (18.8%) died, while 147,619 patients (81.2%) survived.

**Table 1:** Demographic, clinical, and laboratory characteristics of 181,804 patients on hemodialysis based on the dialyzer classification.

Variable	I a	II a	II b	S	*P*-value
*n* (%male)	102,992 (61.4)	64,155 (70.9)	4325 (76.1)	10,332 (57.1)	< 0.0001
Age, years	71.1 ± 11.8	66.4 ± 12.1	63.3 ± 11.9	73.9 ± 11.2	< 0.0001
Vintage, months	60 [27–120]	78 [37–144]	88 [43–165]	54 [24–107]	< 0.0001
Cause of ESKD					< 0.0001
Diabetic nephropathy	39.8	39.7	36.6	39.8	
Chronic glomerulonephritis	28.7	31.6	35.7	27.3	
Nephrosclerosis	12.7	10.8	10.5	13.8	
Others	18.8	17.9	17.2	19.1	
Diabetes mellitus, %	54.6	54.3	48.2	55.7	< 0.0001
Comorbid CVD, %	35.4	31.8	29.1	37.7	< 0.0001
Systolic BP, mmHg	150 ± 25	152 ± 24	152 ± 24	150 ± 26	< 0.0001
Diastolic BP, mmHg	76 ± 14	79 ± 15	81 ± 15	76 ± 15	< 0.0001
Heart rate, bpm	74 ± 13	75 ± 13	76 ± 13	74 ± 13	< 0.0001
Body mass index, kg/m^2^	21.2 ± 3.8	22.4 ± 4.2	22.8 ± 4.2	20.7 ± 3.7	< 0.0001
Serum urea nitrogen, mg/dL	59.8 ± 15.9	61.4 ± 15.4	61.5 ± 14.5	56.4 ± 16.2	< 0.0001
Creatinine, mg/dL	9.3 ± 2.8	10.5 ± 2.7	11.0 ± 2.6	8.4 ± 2.7	< 0.0001
β_2-_microglobulin, mg/L	27.0 ± 7.0	27.2 ± 6.3	27.6 ± 6.4	27.9 ± 8.3	< 0.0001
Kt/V	1.45 ± 0.31	1.49 ± 0.30	1.52 ± 0.31	1.41 ± 0.31	< 0.0001
Serum albumin, g/dL	3.5 ± 0.5	3.6 ± 0.4	3.6 ± 0.4	3.3 ± 0.5	< 0.0001
Hemoglobin, g/dL	10.8 ± 1.3	11.0 ± 1.3	11.1 ± 1.3	10.6 ± 1.4	< 0.0001
C-reactive protein, mg/dL	0.16 [0.06–0.52]	0.14 [0.05–0.42]	0.14 [0.06–0.39]	0.20 [0.07–0.68]	< 0.0001
Calcium, mg/dL	8.7 ± 0.7	8.7 ± 0.7	8.7 ± 0.7	8.5 ± 0.8	< 0.0001
Phosphate, mg/dL	5.1 ± 1.4	5.3 ± 1.5	5.5 ± 1.5	5.1 ± 1.5	< 0.0001
Intact-PTH, pg/mL	126 [69–204]	136 [77–214]	140 [81–222]	123 [66–202]	< 0.0001

*BP* blood pressure, *CVD* cardiovascular disease, *ESKD* end-stage kidney disease, *PTH* parathyroid hormone.

### Predictors of all-cause mortality in 181,804 patients with hemodialysis

The hazard ratios (HRs) for variables assessed as potential predictors of mortality in all patients are presented in Supplementary Table S4. Male gender, advancing age, longer dialysis duration, the presence of DM, and comorbid CVD were identified as significant predictors of mortality. A higher dialysis dose, as indicated by higher single-pool Kt/V and lower β2MG levels, was associated with a lower mortality risk. Lower systolic and diastolic BPs were also associated with a higher mortality risk. Furthermore, poor nutritional status and increased inflammatory status, as indicated by lower hemoglobin levels, higher CRP levels, lower serum albumin levels, and a lower BMI, were associated with a higher mortality rate in patients undergoing HD.

### Associations of the four dialyzer groups with all-cause mortality

**Figure 2:**
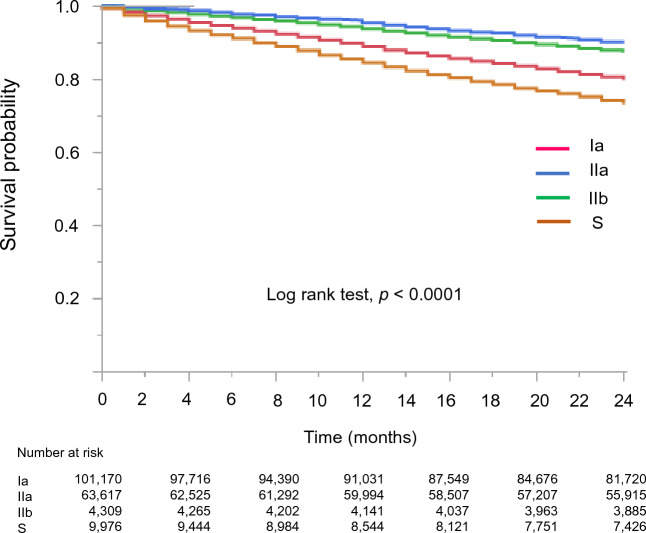
Kaplan–Meier survival curve displaying the rates of all-cause mortality categorized by dialyzer groups.

**Figure 3:**
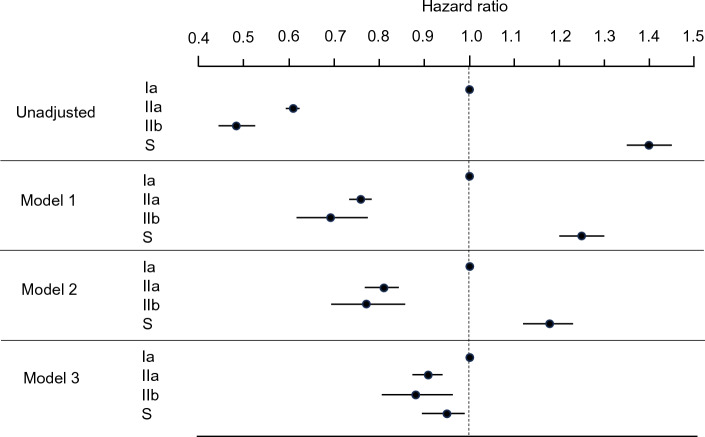
Hazard ratios (HRs) for all-cause mortality in a cohort of 181,804 patients undergoing hemodialysis categorized by dialyzer groups using Cox proportional hazards regression analysis. Circles represent the HR for mortality, and the error bars represent the 95% confidence interval (CI). Model 1 is adjusted for basic factors including age, gender, dialysis vintage, the presence or absence of diabetes mellitus, and the presence or absence of cardiovascular complications. Model 2 is adjusted for dialysis-related factors including Kt/V values, β_2_-microglobulin levels, and systolic and diastolic blood pressure levels, in addition to basic factors. Model 3 is adjusted for basic, dialysis-related, and nutrition- and inflammation-related factors, including body mass index, C-reactive protein, hemoglobin, calcium, phosphate, intact parathyroid hormone, and serum albumin levels.

The Kaplan–Meier analysis revealed a significant variation in survival based on the dialyzer type (log-rank test, *p* < 0.0001; Fig. 2). Compared to the Ia dialyzer group (reference), the S dialyzer group exhibited a higher unadjusted risk for all-cause mortality (HR: 1.39, 95% confidence interval [CI] 1.34–1.45), while the IIa and IIb dialyzer groups showed lower unadjusted risks (HR 0.61, 95% CI 0.59–0.62; HR 0.48, 95% CI 0.44–0.53; Fig. 3, Supplementary Table S5).

The adjusted HRs for all-cause mortality in each group are presented in Fig. 3. After adjusting for basic factors, including age, gender, dialysis duration, history of CVD, and presence or absence of DM, the HRs for the type IIa and IIb dialyzer groups, compared to the type Ia group (reference), were 0.76 (95% CI 0.74–0.78) and 0.69 (95% CI 0.63–0.77), respectively. After adjusting for basic and dialysis-related factors, including Kt/V**,** β2MG levels, and systolic and diastolic BPs, the HRs for the type IIa and IIb groups were 0.81 (95% CI 0.78–0.83) and 0.76 (95% CI 0.69–0.86), respectively. Finally, after adjusting for basic, dialysis-related, and nutrition- and inflammation-related factors, including BMI, hemoglobin, serum albumin, and CRP levels, the type IIa and IIb groups exhibited significantly lower HRs of 0.91 (95% CI 0.87–0.93, *p* < 0.0001) and 0.87 (95% CI 0.78–0.97, *p* = 0.009), respectively (Fig. 3, Supplementary Table S5). The type S dialyzer group demonstrated significantly higher HRs after adjustment for basic and dialysis-related factors than the type Ia dialyzer group. However, it demonstrated a significantly lower HR of 0.95 (95% CI 0.89–0.99, *p* = 0.013) after adjustment for basic, dialysis-related, and nutrition- and inflammation-related factors (Fig. 3, Supplementary Table S5).

### Propensity score-matching analysis

**Figure 4:**
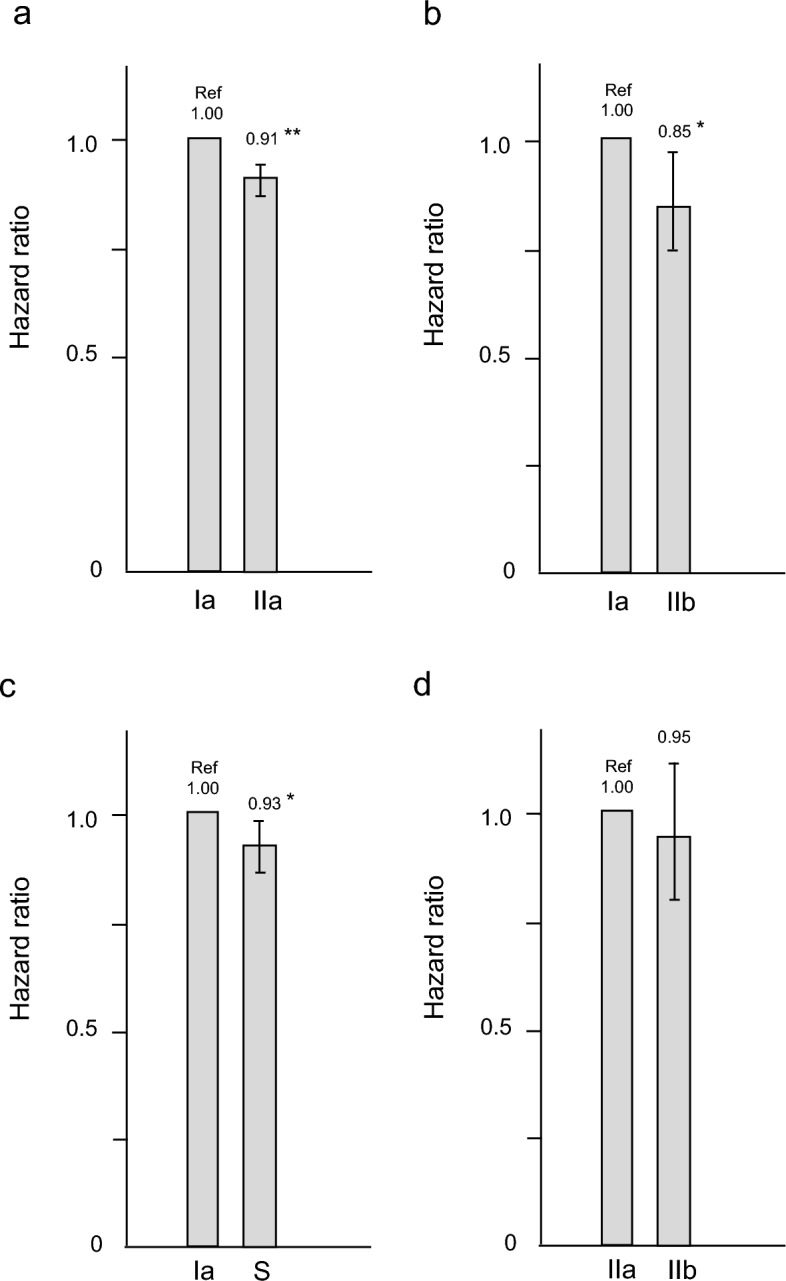
Hazard ratios for all-cause mortality in the four dialyzer groups compared to the reference group after propensity score matching using a Cox proportional hazards regression model. (**a**) Ia group *vs*. IIa group; (**b**) Ia group *vs*. IIb group; (**c**) Ia group *vs*. S group; and (**d**) IIa group vs. the IIb group. *P < 0.05, **p < 0.0001 *vs*. Ia group. Error bars correspond to 95% confidence intervals.

Patients treated with type Ia dialyzers were matched with those treated with other types of dialyzers in a 1:1 ratio according to propensity scores. After PSM, 35,856, 3685, and 7081 patient pairs were matched in the type IIa, IIb, and S dialyzer groups, respectively. Table 2 presents patient characteristics and clinical data at baseline in the type Ia and IIa groups before and after PSM. No significant differences were observed in any of the variables. After PSM, the distributions of patients receiving HD with types I–IV dialyzer in the Ia group were 0.5%, 0.7%, 8.5%, and 90.3%, respectively. As shown in Fig. 4a, compared to the type Ia group, the type IIa group exhibited a lower HR of 0.91 (95% CI 0.87–0.95, *p* < 0.0001). Table 3 summarizes patient characteristics and clinical data at baseline in the type Ia and IIb groups before and after PSM. After PSM, the distributions of patients receiving HD with types I–IV dialyzer in the Ia group were 0.2%, 0.8%, 6.0%, and 93.0%, respectively. Although no significant differences were found in any of the variables, compared to the type Ia group, the type IIb group exhibited a lower HR of 0.85 (95% CI 0.75–0.99, *p* = 0.034; Fig. 4b). Table 4 summarizes patient characteristics and clinical data at baseline in the type Ia and S groups before and after PSM. No significant differences were observed in any of the variables. As shown in Fig. 4c, compared to the type Ia group, the type S group had a lower HR of 0.93 (95% CI 0.87–0.99, *p* = 0.037).

**Table 2:** Comparison of variables before and after propensity score matching between the type Ia and IIa groups.

Variable	Before matching			After matching		
	Ia	IIa	*P*-value	Ia	IIa	*P*-value
*n* (%male)	102,992 (61.4)	64,155 (70.9)	< 0.0001	35,856 (69.4)	35,856 (69.6)	0.559
Age, years	71.1 ± 11.8	66.4 ± 12.1	< 0.0001	67.5 ± 11.8	67.5 ± 11.5	0.701
Vintage, months	60 [27–120]	78 [37–144]	< 0.0001	70 [34–137]	73 [36–135]	0.422
Diabetes mellitus, %	54.6	54.3	0.315	53.6	53.5	0.616
Comorbid CVD, %	35.4	31.8	< 0.0001	35.3	35.4	0.673
Systolic BP, mmHg	150 ± 25	152 ± 24	< 0.0001	152 ± 24	152 ± 24	0.833
Diastolic BP, mmHg	76 ± 14	79 ± 15	< 0.0001	78 ± 14	78 ± 14	0.921
Heart rate, bpm	74 ± 13	75 ± 13	< 0.0001	74 ± 13	74 ± 13	0.467
BMI, kg/m^2^	21.2 ± 3.8	22.4 ± 4.2	< 0.0001	22.1 ± 4.0	22.2 ± 4.0	0.467
Serum UN, mg/dL	59.8 ± 15.9	61.4 ± 15.4	< 0.0001	61.2 ± 15.8	61.6 ± 14.6	0.229
Creatinine, mg/dL	9.3 ± 2.8	10.5 ± 2.7	< 0.0001	11.0 ± 2.9	11.0 ± 2.6	0.628
β2MG, mg/L	27.0 ± 7.0	27.2 ± 6.3	< 0.0001	27.1 ± 6.5	27.1 ± 6.4	0.962
Kt/V	1.45 ± 0.31	1.49 ± 0.30	< 0.0001	1.49 ± 0.29	1.49 ± 0.29	0.748
Serum albumin, g/dL	3.5 ± 0.5	3.6 ± 0.4	< 0.0001	3.6 ± 0.4	3.6 ± 0.4	0.954
Hemoglobin, g/dL	10.8 ± 1.3	11.0 ± 1.3	< 0.0001	10.9 ± 1.2	10.9 ± 1.2	0.932
CRP, mg/dL	0.16 [0.06–0.52]	0.14 [0.05–0.42]	< 0.0001	0.14 [0.06–0.41]	0.14 [0.05–0.40]	0.179

*BMI* body mass index, *BP* blood pressure, *β2MG* β_2_-microglobulin, *CRP* C-reactive protein, *CVD* cardiovascular disease, *UN* urea nitrogen.

**Table 3:** Comparison of variables before and after propensity score matching between the type Ia and IIb groups.

Variable	Before matching			After matching		
	Ia	IIb	*P*-value	Ia	IIb	*P*-value
*n* (%male)	102,992 (61.4)	4325 (76.1)	< 0.0001	3685 (76.5)	3685 (76.5)	0.912
Age, years	71.1 ± 11.8	63.3 ± 11.9	< 0.0001	63.5 ± 12.7	63.2 ± 11.8	0.330
Vintage, months	60 [27–120]	88 [43–165]	< 0.0001	77 [34–158]	86 [42–161]	0.972
Diabetes mellitus, %	54.6	48.2	0.315	49.2	48.9	0.743
Comorbid CVD, %	35.4	29.1	< 0.0001	30.3	30.3	0.501
Systolic BP, mmHg	150 ± 25	152 ± 24	< 0.0001	152 ± 24	152 ± 24	0.249
Diastolic BP, mmHg	76 ± 14	81 ± 15	< 0.0001	80 ± 15	80 ± 15	0.459
Heart rate, bpm	74 ± 13	76 ± 13	< 0.0001	75 ± 13	75 ± 13	0.298
BMI, kg/m^2^	21.2 ± 3.8	22.8 ± 4.2	< 0.0001	22.9 ± 4.3	22.9 ± 4.2	0.833
Serum UN, mg/dL	59.8 ± 15.9	61.5 ± 14.5	< 0.0001	60.7 ± 15.3	60.8 ± 15.2	0.088
Creatinine, mg/dL	9.3 ± 2.8	11.0 ± 2.6	< 0.0001	10.1 ± 2.7	10.2 ± 2.7	0.739
β2MG, mg/L	27.0 ± 7.0	27.6 ± 6.4	< 0.0001	27.1 ± 6.6	27.4 ± 6.4	0.151
Kt/V	1.45 ± 0.31	1.52 ± 0.31	< 0.0001	1.51 ± 0.31	1.52 ± 0.32	0.245
Serum albumin, g/dL	3.5 ± 0.5	3.6 ± 0.4	< 0.0001	3.6 ± 0.4	3.6 ± 0.4	0.546
Hemoglobin, g/dL	10.8 ± 1.3	11.1 ± 1.3	< 0.0001	10.9 ± 1.2	10.9 ± 1.2	0.950
CRP, mg/dL	0.16 [0.06–0.52]	0.14 (0.06–0.39)	< 0.0001	0.15 [0.06–0.44]	0.14 [0.06–0.39]	0.674

*BMI* body mass index, *BP* blood pressure, *β2MG* β_2_-microglobulin, *CRP* C-reactive protein, *CVD* cardiovascular disease, *UN* urea nitrogen.

**Table 4:** Comparison of variables before and after propensity score matching between the type Ia and S groups.

Variable	Before matching			After matching		
	Ia	S	*P*-value	Ia	S	*P*-value
*n* (%male)	102,992 (61.4)	10,332 (57.1)	< 0.0001	7081 (58.2)	7081 (58.0)	0.785
Age, years	71.1 ± 11.8	73.9 ± 11.2	< 0.0001	73.7 ± 11.3	73.7 ± 11.1	0.882
Vintage, months	60 [27–120]	54 [24–107]	< 0.0001	54 [24–106]	54 [25–105]	0.272
Diabetes mellitus, %	54.6	55.7	0.034	53.5	53.7	0.813
Comorbid CVD, %	35.4	37.7	< 0.0001	40.2	41.0	0.365
Systolic BP, mmHg	150 ± 25	150 ± 26	0.184	150 ± 25	150 ± 25	0.504
Diastolic BP, mmHg	76 ± 14	76 ± 15	< 0.0001	75 ± 14	75 ± 14	0.139
Heart rate, bpm	74 ± 13	74 ± 13	0.023	73 ± 13	74 ± 13	0.168
BMI, kg/m^2^	21.2 ± 3.8	20.7 ± 3.7	< 0.0001	20.8 ± 3.8	20.8 ± 3.7	0.534
Serum UN, mg/dL	59.8 ± 15.9	56.4 ± 16.2	< 0.0001	57.1 ± 16.1	57.2 ± 15.9	0.127
Creatinine, mg/dL	9.3 ± 2.8	8.4 ± 2.7	< 0.0001	8.6 ± 2.7	8.5 ± 2.7	0.172
β2MG, mg/L	27.0 ± 7.0	27.9 ± 8.3	< 0.0001	27.5 ± 7.7	27.5 ± 8.3	0.883
Kt/V	1.45 ± 0.31	1.41 ± 0.31	< 0.0001	1.43 ± 0.31	1.42 ± 0.31	0.351
Serum albumin, g/dL	3.5 ± 0.5	3.3 ± 0.5	< 0.0001	3.3 ± 0.5	3.3 ± 0.5	0.590
Hemoglobin, g/dL	10.8 ± 1.3	10.6 ± 1.4	< 0.0001	10.7 ± 1.3	10.7 ± 1.3	0.617
CRP, mg/dL	0.16 [0.06–0.52]	0.20 [0.07–0.68]	< 0.0001	0.21 [0.08–0.68]	0.19 [0.07–0.63]	0.598

*BMI* body mass index, *BP* blood pressure, *β2MG* β_2_-microglobulin, *CRP* C-reactive protein, *CVD* cardiovascular disease, *UN* urea nitrogen.

Patients receiving HD with type IIa dialyzers were matched with those receiving HD with type IIb dialyzers at a 1:1 ratio according to propensity scores. After PSM, 2555 patient pairs were matched in the type IIb dialyzer group. Table 5 presents the baseline demographic and clinical characteristics of the type IIa and IIb groups before and after PSM. No considerable differences were observed in any of the variables. As shown in Fig. 4d, the type IIa and IIb groups did not significantly differ in terms of mortality (HR 0.95 [95% CI 0.80–1.12], *p* = 0.55).

**Table 5:** Comparison of variables before and after propensity score matching between the type IIa and IIb groups.

Variables	Before matching			After matching		
	II a	II b	*P*-value	II a	II b	*P*-value
*n* (%male)	64,155 (70.9)	4325 (76.1)	< 0.0001	2555 (75.6)	2555 (76.1)	0.695
Age, years	66.4 ± 12.1	63.3 ± 11.9	< 0.0001	63.3 ± 12.2	63.2 ± 11.8	0.612
Vintage, months	78 [37–144]	88 [43–165]	< 0.0001	88 [45–161]	89 [45–163]	0.891
Diabetes mellitus, %	54.3	48.2	< 0.0001	49.5	48.1	0.261
Comorbid CVD, %	31.8	29.1	0.0002	33.3	31.6	0.818
Systolic BP, mmHg	152 ± 24	152 ± 24	0.537	152 ± 23	152 ± 24	0.402
Diastolic BP, mmHg	79 ± 15	81 ± 15	< 0.0001	81 ± 15	81 ± 15	0.268
Heart rate, bpm	75 ± 13	76 ± 13	< 0.0001	77 ± 13	77 ± 13	0.616
BMI, kg/m^2^	22.4 ± 4.2	22.8 ± 4.2	< 0.0001	22.8 ± 4.2	22.8 ± 4.1	0.729
Serum UN, mg/dL	61.4 ± 15.4	61.5 ± 14.5	0.573	61.7 ± 14.5	61.6 ± 14.3	0.746
Creatinine, mg/dL	10.5 ± 2.7	11.0 ± 2.6	< 0.0001	10.9 ± 2.6	11.0 ± 2.5	0.483
β2MG, mg/L	27.2 ± 6.3	27.6 ± 6.4	0.0009	27.5 ± 6.5	27.5 ± 6.2	0.904
Kt/V	1.49 ± 0.30	1.52 ± 0.31	< 0.0001	1.54 ± 0.31	1.54 ± 0.31	0.592
Serum albumin, g/dL	3.6 ± 0.4	3.6 ± 0.4	< 0.0001	3.6 ± 0.3	3.6 ± 0.3	0.190
Hemoglobin, g/dL	11.0 ± 1.3	11.1 ± 1.3	< 0.0001	11.1 ± 1.2	11.2 ± 1.2	0.589
CRP, mg/dL	0.14 [0.05–0.42]	0.14 [0.06–0.39]	0.0008	0.14 [0.05–040]	0.14 [0.06–0.38]	0.784

*BMI* body mass index, *BP* blood pressure, *β2MG* β_2_-microglobulin, *CRP* C-reactive protein, *CVD* cardiovascular disease, *UN* urea nitrogen.

## Discussion

This observational cohort study provides novel evidence supporting the improved survival associated with the current Japanese dialyzer classification. The study analyzed data from a large-scale registry of 181,804 Japanese patients on HD, with a two-year follow-up period. The results demonstrate a significant association between the use of type IIa, IIb, and S dialyzers and lower all-cause mortality. Mortality rates were compared among the four dialyzer types, taking into consideration predictive factors and adjusting for confounders. After adjusting for predictive factors and using PSM, the HR was significantly lower in the type IIa, IIb, and S dialyzer groups than in the type Ia group (reference). Furthermore, the study revealed the superiority of super high-flux membrane dialyzers, as indicated by a higher β2MG clearance rate regardless of albumin SC. The study’s major strengths include its large sample size and inclusion of all current dialyzer types. Notably, this study is the first to suggest a potential reduction in mortality risk among patients on HD using super high-flux dialyzers, defined as those with a β2MG clearance rate of ≥ 70 mL/min.

Recent studies have focused on the removal of not only small-middle molecules, such as β2MG (molecular weight: 11.8 kDa), but also large-middle molecules, such as α1-microglobulin (molecular weight: 33.0 kDa), in patients on dialysis to improve prognosis^12,13^. The effectiveness of removing middle molecules depends on both dialyzer permeability and treatment modality. Therefore, online HDF using high-flux dialyzers is considered a more efficient treatment modality compared to HD using low-flux and high-flux dialyzers. In particular, high-volume post-dilution online HDF, which involves a convective volume of at least 23 L/session, allows for greater removal of uremic toxins and may lead to improved outcomes^14,15^. This treatment offers the best clearance of small and middle molecules and is widely used in Japan and some European countries. However, online HDF may not be suitable for all patients on maintenance HD and is not widely available in many countries. Considering the limitations of high-volume post-dilution online HDF, HD with a novel medium cutoff (MCO) type of dialyzer that has a larger pore size than standard high-flux dialyzers could potentially enhance the removal of medium- and large-middle molecules^16^. Super high-flux dialyzers exhibit distinct features, encompassing not only a higher ultrafiltration coefficient but also a higher β2MG clearance rate^17^. As super high-flux dialyzers have larger pores than high-flux membranes, they possess the capacity to remove molecules of varying sizes, spanning small to large, including those categorized as large-middle molecules, as well as trace amounts of albumin^18,19^. The optimal pore size should mitigate albumin loss exceeding 3 g per session during standard HD procedures in Japan, characterized by a blood flow rate of 200 mL/min and a dialysate flow rate of 500 mL/min^7,19^. Notably, super high-flux dialyzers or protein-leaking dialyzers have demonstrated noninferiority to high-volume post-dilution online HDF in the removal of protein-bound and middle-molecule toxins^20–22^, making them an option for patients on long-term HD. However, these previous studies were short-term, focusing on solute clearance without exploring broader outcomes. The type Ia group in the present study included approximately 11% of old types I, II, and III dialyzers, defined as β2MG clearance of < 50 mL/min, which might have contributed to the inferiority of the type Ia group to type IIa and IIb groups. This study asserts the superiority of dialyzers with a β2MG clearance rate of 70 mL/min or higher, even within the super high-flux category. Super-high flux dialyzers were more effective in eliminating β2MG and α1-microglobulin or uremic substances with similar molecular weights than type I dialyzers. Hence, they might be associated with a better prognosis. However, further investigation should be performed to validate super high-flux dialyzers as the removal rate of uremic substances in each group could not be evaluated.

Super high-flux dialyzers demonstrate a reduced mortality risk when compared to both low-flux dialyzers (defined by β2MG clearance rate < 10 mL/min) and high-flux dialyzers (defined by β2MG clearance rate ranging from 10 to < 50 mL/min)^8,9^. In Europe, where the blood flow rate (QB) surpasses that in Japan, low-flux membranes are characterized by a β2MG clearance of < 10 mL/min with an albumin SC of 0. Meanwhile, high-flux membranes are characterized by a β2MG clearance of > 20 mL/min with an albumin SC of < 0.01^23^. In Europe, high-volume (16–26 L) post-dilution online HDF using low-permeability membranes of albumin has been conducted with limited albumin leakage, not exceeding 3.4 g/session^24^ or 5 g/session in a convection volume of 23 L/session/1.73 m^2^^25^. Despite the ongoing debate regarding acceptable albumin leakage during HD or HDF, patients treated with high albumin leakage dialyzers have reported better survival rates than those treated with low albumin leakage dialyzers, evident in both super high-flux HD and online HDF^26^. Furthermore, survival rates remain comparable between patients on online HDF and super high-flux HD with similar levels of albumin leakage^26^. Consequently, the deliberate promotion of albumin leakage in both online HDF and super high-flux HD is considered significant, as high albumin leakage dialyzers, effectively eliminating uremic toxins with large molecules, are associated with improved mortality outcomes. Notably, in this study, the superiority of type IIb dialyzers over type IIa dialyzers could not be confirmed. Type IIb dialyzers with enhanced solute removal capabilities, including large molecules to mitigate hypoalbuminemia, may be beneficial in patients without malnutrition or inflammation. Further studies are required to substantiate the hypothesis that dialyzers with higher albumin leakage contribute to improved mortality outcomes in patients undergoing HD.

In this study, it was found that type S dialyzers, specifically those with ethylene–vinyl alcohol co-polymer (EVOH) and polymethyl methacrylate (PMMA) membranes, demonstrated a better prognosis compared to other types. EVOH membranes, unlike other types, do not require hydrophilic agents such as polyvinylpyrrolidone and have low plasma protein adsorption^27^. Furthermore, they have been reported to induce less platelet activation and reactive oxygen species production through neutrophil activation, indicating excellent biocompatibility^28,29^. PMMA membranes, on the other hand, have a uniform symmetrical structure with relatively large pores and broad-type fractionation characteristics, making them effective in removing large molecules similar to albumin^30^. Furthermore, due to the absence of a hydrophilic agent like polyvinylpyrrolidone, type S dialyzers have protein adsorption properties, enabling the adsorption and removal of middle and large molecules that are particularly difficult to permeate through membranes. PMMA membranes, in particular, are capable of adsorbing and removing high-molecular-weight pathogenic substances, such as cytokines and proteins, that cannot be effectively eliminated by other dialysis membranes^31^. They have shown effectiveness in improving pruritus and maintaining dry weight in elderly patients on dialysis^31–33^. In addition, a nationwide cohort study conducted in 2009 reported that PMMA membrane dialyzers may improve prognosis compared to polysulfone membrane dialyzers in Japanese patients undergoing HD^34,35^. Patients treated with type S dialyzers tend to be elderly and predominantly female, with higher rates of comorbid CVD, a lower BMI, and lower serum albumin levels. Initially, the mortality rate in the type S group was significantly higher than that in the type Ia group in the unadjusted model. However, after accounting for nutrition- and inflammation-related factors and conducting PSM analysis, the HR for all-cause mortality in the type S group was significantly lower than that in the type Ia group. Therefore, type S dialyzers, with their characteristics of minimal albumin loss, high solute permeability (particularly for uremic toxins with molecular weights of 10–30 kDa), and high biocompatibility, may be suitable for malnourished elderly patients.

This study has several limitations that should be considered. First, the number of patients differed among the four groups, which is inherent to the annual survey and observational cohort study design. In addition, the number of patients treated with type Ib dialyzers was only 74, and they were excluded from the analysis. Further, information on whether the patients have been previously treated with the same type of dialyzers during the observation period could not be collected. However, after conducting PSM analysis, the superiority of type IIa, IIb, and S dialyzers was confirmed. Second, information regarding the effects of facility protocols or the practice patterns of the dialysis unit was not available. However, reimbursement for dialysis sessions including dialyzers is similar regardless of economic status because the insurance system is universal in Japan. Therefore, the type of dialyzer used is based on the discretion of the physicians at each facility. However, these factors can be potential confounders and may contribute to variations in mortality rates among different centers due to differences in center practices and patient populations. Third, this study included patients who have dialysis vintage for several years, indicating a selected group of survivors. Cardiovascular disease is the leading cause of mortality among Japanese patients on dialysis. Meanwhile, infection is the most common cause of mortality in patients on incident dialysis^36^. Therefore, further investigation should be performed to validate the effect of super-high flux dialyzers on improving prognosis even in patients on incident dialysis. Finally, patients treated with HDF were excluded from the present study to eliminate modality bias. However, the number of patients receiving pre-dilution online HDF has been increasing in Japan, and it is considered to be a highly efficient technique for using high-flux membranes. It achieves higher clearance of small solutes such as urea and small-, middle-, and large-middle molecules like β2MG and α_1_-microglobulin compared to high-flux HD^37^. Therefore, further clinical trials are required to investigate the impact of this modality on mortality outcomes.

In conclusion, this large national cohort study of Japanese patients undergoing dialysis has provided valuable insights into the association between dialyzer type, classified by β2MG clearance and albumin SC, and the two-year mortality rate. These findings suggest that super high-flux dialyzers with a β2MG clearance rate of more than 70 mL/min may be beneficial for patients undergoing HD, regardless of albumin SC. In addition, type S dialyzers may be beneficial for elderly and malnourished patients on dialysis. Further randomized controlled studies are warranted to determine whether the higher β2MG clearance of super high-flux dialyzers truly improves outcomes for patients on HD.

### Supplementary Information

Supplementary Legends.

Supplementary Figure S1.

Supplementary Table S1.

Supplementary Table S2.

Supplementary Table S3.

Supplementary Table S4.

Supplementary Table S5.

## Data Availability

The data used in this study are available from the corresponding author.
