# Supplementary material for: Current dialyzer classification in Japan and mortality risk in patients undergoing hemodialysis
Source: Sci Rep. 2024 May 4;14:10272. doi: 10.1038/s41598-024-60831-y (PMC11069571; doi:10.1038/s41598-024-60831-y)
Supplement: Supplementary file 1 — Supplementary Legends. [file 41598_2024_60831_MOESM1_ESM.docx]

Supplementary Figure S1. Schematic diagram depicting the dialyzer classification in Japan since 2013

β2MG, β_2_-microglobulin
