# Supplementary figures and images for: Current dialyzer classification in Japan and mortality risk in patients undergoing hemodialysis
Source: Sci Rep. 2024 May 4;14:10272. doi: 10.1038/s41598-024-60831-y (PMC11069571; doi:10.1038/s41598-024-60831-y)

## Slide 1
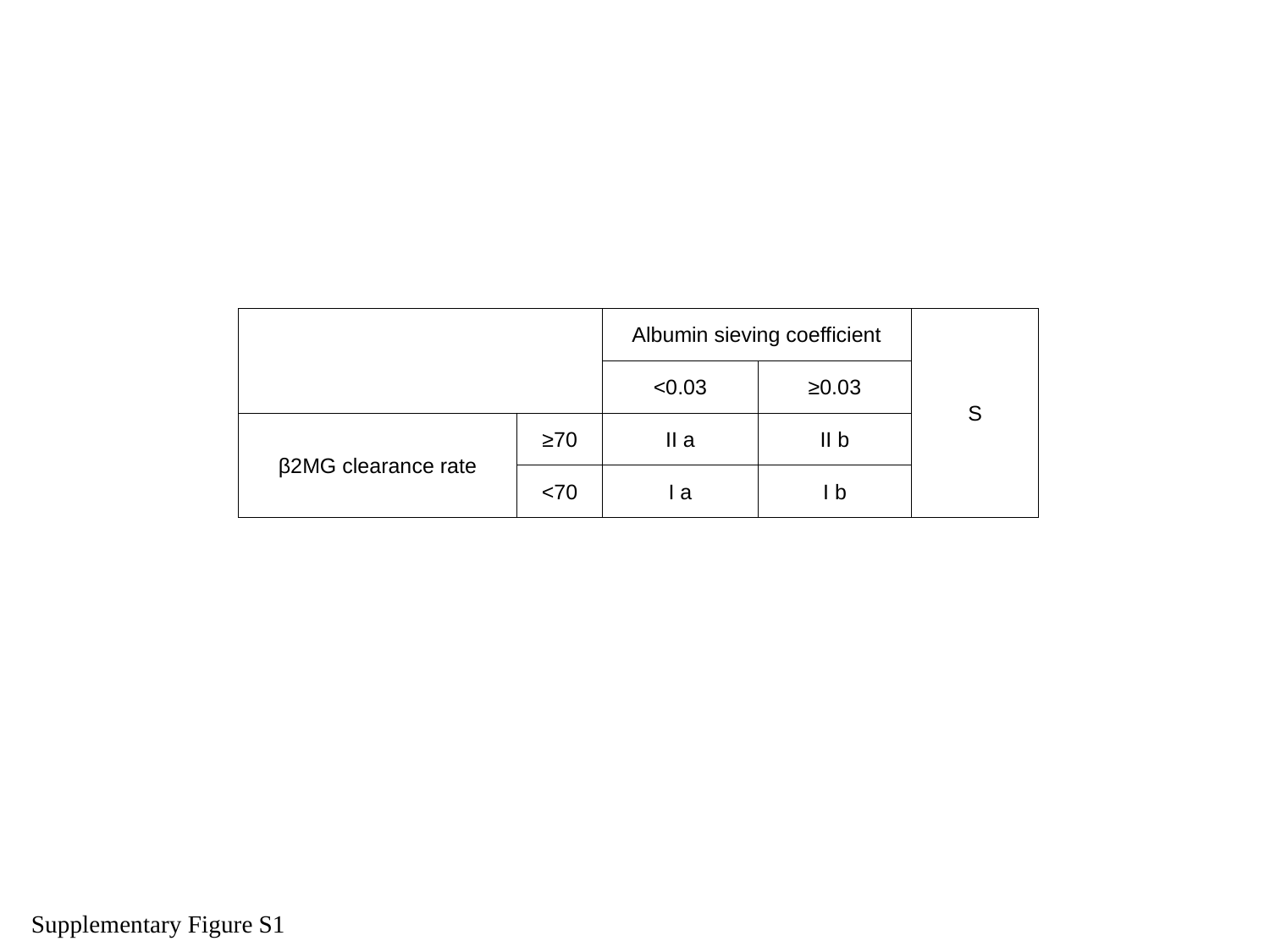

| | | Albumin sieving coefficient | | S |
| --- | --- | --- | --- | --- |
| | | <0.03 | ≥0.03 | |
| β2MG clearance rate | ≥70 | II a | II b | |
| | <70 | I a | I b | |
Supplementary Figure S1

Supplement: Supplementary file 2 — Supplementary Figure S1. [file 41598_2024_60831_MOESM2_ESM.pptx]
