# Supplementary material for: Current dialyzer classification in Japan and mortality risk in patients undergoing hemodialysis
Source: Sci Rep. 2024 May 4;14:10272. doi: 10.1038/s41598-024-60831-y (PMC11069571; doi:10.1038/s41598-024-60831-y)
Supplement: Supplementary file 3 — Supplementary Table S1. [file 41598_2024_60831_MOESM3_ESM.docx]

Supplementary Table S1. Old dialyzer classification based on β_2_-microglobulin clearance in Japan until 2012

| Old classification | International classification | β2MG clearance (mL/min) | UN clearance (mL/min) | UFR (mL/h/mmHg) |
| --- | --- | --- | --- | --- |
| I | Low-flux | <10 | ≥125 | <15 |
| II | High-flux | ≥10–30 | ≥150 | ≥15 |
| III | High-flux | ≥30–50 | ≥150 | ≥15 |
| IV | High-flux | ≥50–70 | ≥150 | ≥50 |
| V | Super-high flux | ≥70 | ≥150 | ≥50 |

β2MG, β2-microglobulin; UFR, ultrafiltration rate; UN, urea nitrogen.
