# Supplementary material for: Current dialyzer classification in Japan and mortality risk in patients undergoing hemodialysis
Source: Sci Rep. 2024 May 4;14:10272. doi: 10.1038/s41598-024-60831-y (PMC11069571; doi:10.1038/s41598-024-60831-y)
Supplement: Supplementary file 4 — Supplementary Table S2. [file 41598_2024_60831_MOESM4_ESM.docx]

Supplementary Table S2. Current dialyzer classification based on urea and β_2_-microglobulin clearance and albumin sieving coefficient in Japan since 2013

| Type of dialyzers | I | | II | | S |
| --- | --- | --- | --- | --- | --- |
|  | Ia | Ib | IIa | IIb |  |
| Measurement condition* |  | | | | |
| Surface area, m^2^ | 1.5 | | | | |
| Blood flow rate, mL/min | 200 ± 4 | | | | |
| Dialysate flow rate, mL/min | 500 ± 15 | | | | |
| Substitution flow rate, mL/min | 15 ± 1 | | | | |
| Performance standards |  | |  | |  |
| Urea clearance (mL/min) | ≥150 | | ≥185 | | 150 |
| β2MG clearance (mL/min) | <70 | | ≥70 | | 0 |
| Albumin sieving coefficient | <0.03 | ≥0.03 | <0.03 | ≥0.03 |  |

*The average temperature of the bovine blood and dialysate solution in the storage tank should be 37°C ± 1°C. Measurements were taken 60 and 240 min after the start of the experiment. β2MG, β_2_-microglobulin.
