# Supplementary material for: Current dialyzer classification in Japan and mortality risk in patients undergoing hemodialysis
Source: Sci Rep. 2024 May 4;14:10272. doi: 10.1038/s41598-024-60831-y (PMC11069571; doi:10.1038/s41598-024-60831-y)
Supplement: Supplementary file 5 — Supplementary Table S3. [file 41598_2024_60831_MOESM5_ESM.docx]

Supplementary Table S3. Characteristics of the dialyzers used in this study

| Material | Company | Japanese brand name | Surface area (m^2^) | UFR ^a^ (mL/mmHg/h) | Clearance (mL/min) ^b^ | | | | |  | SC ^c^ | Old classification | Current classification |
| --- | --- | --- | --- | --- | --- | --- | --- | --- | --- | --- | --- | --- | --- |
|  |  |  |  |  | UN | Cr | P | Vit B_12_ | β2MG |  | Alb |  |  |
| CTA | Nipro Co. | FB-EG eco | 1.5 | 12.8 | 191 | 180 | 166 | 100 | 8 |  | 0 | I | Ia |
|  |  | FB-G eco | 1.5 | 17.7 | 191 | 180 | 166 | 116 | 20 |  | 0 | II | Ia |
|  |  | FB-E eco | 1.5 | 20.5 | 191 | 181 | 169 | 121 | 20 |  | 0 | II | Ia |
|  |  | FB-UP eco | 1.5 | 42.3 | 197 | 191 | 181 | 142 | ≥10,<30 |  | <0.01 | III | Ia |
|  |  | FB-P eco | 1.5 | 25.4 | 193 | 184 | 172 | 125 | ≥30, <50 |  | 0.01 | III | Ia |
|  |  | FB-U eco | 1.5 | 29.8 | 196 | 188 | 182 | 133 | ≥30, <50 |  | 0.01 | III | Ia |
|  |  | FB-F eco | 1.5 | 37.1 | 198 | 190 | 189 | 144 | ≥30, <50 |  | 0.01 | III | Ia |
|  |  | FB-UH eco | 1.5 | 50.1 | 198 | 193 | 192 | 154 | ≥30, <50 |  | 0.01 | III | Ia |
|  |  | FB-Pβ eco | 1.5 | 58.4 | 198 | 192 | 190 | 148 | 53 |  | 0.01 | IV | Ia |
|  |  | FB-Uβ eco | 1.5 | 60.1 | 198 | 192 | 191 | 149 | 65 |  | 0.01 | IV | Ia |
|  |  | FB-Fα eco | 1.5 | 49.6 | 196 | 191 | 184 | 148 | ≥30, 50 |  | 0.05 | III | Ib |
|  |  | FB-FH eco | 1.5 | 66.9 | 198 | 194 | 193 | 155 | ≥50, <70 |  | 0.05 | IV | Ib |
|  |  | FB-UHα eco | 1.5 | 65.4 | 199 | 198 | 191 | 161 | ≥70 |  | 0.02 | V | IIa |
|  |  | FB-UHβ eco | 1.5 | 70.3 | 199 | 198 | 192 | 165 | ≥70 |  | 0.04 | V | IIa |
|  |  | FA-F eco | 1.5 | 62.0 | 199 | 194 | 189 | 157 | ≥70 |  | 0.02 | V | IIa |
| EVOH | Kawasumi Laboratories Inc. | KF | 1.5 | 8.7 | 179 | 157 | 133 | 80 | 17.5 |  | 0.02 | I | S |
|  |  | kf-m | 1.5 | 11.5 | 175 | 155 | 133 | 85 | 16.6 |  | 0.03 | II | S |
|  |  | KF-C | 1.5 | 11.1 | 181 | 165 | 143 | 93 | 44.6 |  | 0.04 | II | S |
| PEPA | Nikkiso Co. | FLX | 1.5 | 54.0 | 192 | 185 | 181 | 154 | <70 |  | 0.02 | IV | Ia |
|  |  | FDX | 1.5 | 54.0 | 190 | 181 | 174 | 136 | <70 |  | 0.01 | IV | Ia |
|  |  | FDY | 1.5 | 54.0 | 190 | 181 | 174 | 136 | <70 |  | 0.02 | IV | Ia |
|  |  | FDW | 1.5 | 57.0 | 190 | 182 | 175 | 138 | >70 |  | 0.01 | V | IIa |
|  |  | FDZ | 1.5 | 59.0 | 190 | 182 | 175 | 140 | >70 |  | 0.04 | V | IIb |
| PES | JMS　Co. | BP-N | 1.5 | 43.0 | 190 | 178 | NA | NA | 75 |  | 0.01 | V | IIa |
|  | Nipro Co. | PES-Gα eco | 1.5 | 31.2 | 197 | 192 | 183 | 157 | ≥50, <70 |  | <0.01 | IV | Ia |
|  |  | PES-Eα eco | 1.5 | 32.7 | 197 | 192 | 183 | 158 | 65 |  | <0.01 | IV | Ia |
|  |  | PES-SGαeco | 1.5 | 64.8 | 197 | 195 | 188 | 159 | ≥70 |  | 0.01 | V | IIa |
|  |  | PES-SEα eco | 1.5 | 43.5 | 199 | 198 | 190 | 166 | ≥70 |  | 0.01 | V | IIa |
|  |  | PES-Sα eco | 1.5 | 43.7 | 199 | 198 | 187 | 160 | ≥70 |  | 0.01 | V | IIa |
|  |  | PES-Dα eco | 1.5 | 49.1 | 199 | 198 | 187 | 161 | 77 |  | 0.02 | V | Iib |
|  |  | PES-DSα eco | 1.5 | 63.7 | 199 | 198 | 191 | 162 | ≥70 |  | 0.04 | V | IIb |
|  |  | PES-Mα eco | 1.5 | 14.1 | 194 | 189 | 170 | 118 | <10 |  | <0.01 | I | Ia |
|  |  | PES-Kα eco | 1.5 | 51.9 | 197 | 191 | 181 | 142 | ≥30, <50 |  | <0.01 | III | Ia |
| PMMA | Toray Co. | NF-U | 1.6 | 30.0 | 190 | 172 | 162 | 112 | ≥30, <50 |  | 0.003 | III | S |
|  |  | BK-U | 1.6 | 31.0 | 187 | 169 | NA | 108 | 51 |  | 0.03 | III | S |
|  |  | NF-H | 1.6 | 35.0 | 193 | 180 | 172 | 126 | ≥50, <70 |  | 0.009 | IV | S |
|  |  | BG-PQ | 1.6 | 38.0 | 191 | 174 | 164 | 114 | 55 |  | 0.009 | IV | S |
|  |  | B3 | 1.6 | 8.7 | 188 | 167 | 128 | 88 | <10 |  | 0 | I | S |
| PS | Fresenius Medical Care Japan | FX-CorDiax | 1.4 | 47.0 | 196 | 190 | 184 | 149 | >70 |  | 0.004 | V | IIa |
|  |  | FX | 1.4 | 46.0 | 198 | 182 | 172 | 127 | 68.1 |  | 0.005 | IV | Ia |
|  |  | FX-S | 1.4 | 70.0 | 196 | 182 | 178 | 132 | >70 |  | 0.026 | V | IIa |
|  | Asahi Kasei-Kuraray Medical Co., Ltd. | APS-E | 1.5 | 66.0 | 190 | 181 | 175 | 138 | 74 |  | 0.04 | V | IIb |
|  |  | APS-EL | 1.5 | 55.0 | 187 | 184 | 173 | 136 | ≥70 |  | 0.02 | V | IIa |
|  |  | APS-EA | 1.5 | 56.0 | 197 | 197 | 186 | 145 | ≥70 |  | 0.02 | V | IIa |
|  |  | APS-MA | 1.5 | 56.0 | 196 | 194 | 183 | 142 | ≥30, <50 |  | <0.01 | III | Ia |
|  |  | APS-MD | 1.5 | 52.0 | 187 | 184 | 172 | 133 | ≥50, <70 |  | <0.01 | IV | Ia |
|  |  | APS-SA | 1.5 | 63.0 | 196 | 191 | 185 | 142 | 68 |  | 0.002 | IV | Ia |
|  |  | APS-UA | 1.5 | 42.0 | 196 | 191 | 185 | 140 | 48 |  | <0.002 | III | Ia |
|  |  | VPS-HA | 1.5 | 63.0 | 196 | 190 | 182 | 138 | 62 |  | 0.002 | IV | Ia |
|  | Toray Co. | NV-S | 1.5 | 45.0 | 195 | 186 | 183 | 144 | ≥50, <70 |  | 0.003 | IV | Ia |
|  |  | NV-U | 1.5 | 45.0 | 196 | 189 | 187 | 150 | ≥50, <70 |  | 0.005 | IV | Ia |
|  |  | NV-X | 1.5 | 45.0 | 197 | 192 | 190 | 158 | ≥70 |  | 0.008 | V | IIa |

All data were provided by the manufacturer. β2MG, β2-microglobulin; Cr, creatinine; CTA, cellulose triacetate; EVOH, ethylene-vinyl alcohol copolymer; PEPA, polyester polymer alloy; PES, polyethersulfone; PMMA, polymethylmethacrylate; PS, polysulfone; SC, sieving coefficient; UFR, ultrafiltration rate; UN, urea nitrogen. a. UFR was measured under the following conditions using bovine blood: Hematocrit = 32±2%, Total protein = 6.0±0.5g/dL, blood flow rate = 200mL/min, dialysis fluid flow rate = 0mL/min, and ultrafiltration rate = 10.0mL/min/m^2^. b. Clearances were measured under the following conditions: blood flow rate = 200 mL/min, dialysis fluid flow rate = 500 mL/min, and ultrafiltration rate = 0 mL/min/m^2^. c. Sieving coefficient was measured under the following conditions using bovine serum: blood flow rate = 200 mL/min and ultrafiltration rate = 10mL/min/m^2^.
