# Supplementary material for: Current dialyzer classification in Japan and mortality risk in patients undergoing hemodialysis
Source: Sci Rep. 2024 May 4;14:10272. doi: 10.1038/s41598-024-60831-y (PMC11069571; doi:10.1038/s41598-024-60831-y)
Supplement: Supplementary file 6 — Supplementary Table S4. [file 41598_2024_60831_MOESM6_ESM.docx]

Supplementary Table S4. Hazard ratios and 95% confidence intervals of variables assessed as potential predictors of mortality in 181,804 patients

| Variables | HR | 95% CI | P value |
| --- | --- | --- | --- |
| Sex |  |  |  |
| Male | 1.000 | Reference | - |
| Female | 0.964 | 0.942–0.987 | 0.002 |
| Age, years | 1.062 | 1.061–1.063 | <0.0001 |
| Dialysis vintage, years |  |  |  |
| <2 | 0.841 | 0.812–0.871 | <0.0001 |
| ≥2–5 | 1.000 | Reference | - |
| ≥5–10 | 1.102 | 1.070–1.114 | <0.0001 |
| ≥10–15 | 1.115 | 1.075–1.156 | <0.0001 |
| ≥15–20 | 0.971 | 0.925–1.017 | 0.213 |
| ≥20 | 0.912 | 0.869–0.955 | <0.0001 |
| Primary kidney disease |  |  |  |
| Glomerulonephritis | 1.000 | Reference | - |
| Diabetic nephropathy | 1.323 | 1.287–1.360 | <0.0001 |
| Nephrosclerosis | 1.365 | 1.315–1.417 | <0.0001 |
| Others | 1.206 | 1.166–1.248 | <0.0001 |
| Diabetes mellitus |  |  |  |
| No | 1.000 |  |  |
| Yes | 1.259 | 1.229–1.289 | <0.0001 |
| Comorbid CVD |  |  |  |
| No | 1.000 | Reference | - |
| Yes | 1.791 | 1.752–1.831 | <0.0001 |
| Body mass index, kg/m^2^ |  |  |  |
| <16 | 3.139 | 3.005–3.280 | <0.0001 |
| ≥16–18 | 1.891 | 1.822–1.961 | <0.0001 |
| ≥18–20 | 1.307 | 1.262–1.353 | <0.0001 |
| ≥20–22 | 1.000 | Reference | - |
| ≥22–24 | 0.788 | 0.756–0.821 | <0.0001 |
| ≥24–26 | 0.642 | 0.609–0.677 | <0.0001 |
| ≥26–28 | 0.587 | 0.548–0.628 | <0.0001 |
| ≥28 | 0.511 | 0.477–0.547 | <0.0001 |
| Serum albumin level, g/dL |  |  |  |
| <2.5 | 11.150 | 10.67–11.66 | <0.0001 |
| ≥2.5–3.0 | 5.432 | 5.251–5.618 | <0.0001 |
| ≥3.0–3.5 | 2.218 | 2.159–2.279 | <0.0001 |
| ≥3.5–4.0 | 1.000 | Reference | - |
| ≥4.0 | 0.553 | 0.526–0.582 | <0.0001 |
| C-reactive protein level |  |  |  |
| 1 mg/dL increase | 1.297 | 1.289–1.305 | <0.0001 |
| Hemoglobin level |  |  |  |
| 1 g/dL increase | 0.792 | 0.785–0.799 | <0.0001 |
| Systolic BP, mmHg |  |  |  |
| <100 | 2.595 | 2.434–2.767 | <0.0001 |
| ≥100–120 | 1.555 | 1.492–1.619 | <0.0001 |
| ≥120–140 | 1.000 | Reference | - |
| ≥140–160 | 0.836 | 0.810–0.862 | <0.0001 |
| ≥160–180 | 0.831 | 0.803–0.859 | <0.0001 |
| ≥180 | 0.876 | 0.841–0.912 | <0.0001 |
| Diastolic BP, mmHg |  |  |  |
| <60 | 2.443 | 2.353–2.537 | <0.0001 |
| ≥60–70 | 1.557 | 1.504–1.612 | <0.0001 |
| ≥70–80 | 1.199 | 1.159–1.240 | <0.0001 |
| ≥80–90 | 1.000 | Reference | - |
| ≥90–100 | 0.882 | 0.843–0.922 | <0.0001 |
| ≥100 | 0.862 | 0.813–0.913 | <0.0001 |
| Heart rate, bpm |  |  |  |
| <60 | 1.182 | 1.136–1.231 | <0.0001 |
| ≥60–70 | 1.047 | 1.015–1.079 | 0.003 |
| ≥70–80 | 1.000 | Reference | - |
| ≥80–90 | 1.053 | 1.018–1.088 | 0.0026 |
| ≥90–100 | 1.121 | 1.073–1.172 | <0.0001 |
| ≥100 | 1.411 | 1.331–1.497 | <0.0001 |
| β_2_-microglobulin level, mg/L |  |  |  |
| <15 | 0.863 | 0.795–0.936 | 0.0003 |
| ≥15–20 | 0.857 | 0.814–0.902 | <0.0001 |
| ≥20–25 | 0.961 | 0.927–0.996 | 0.029 |
| ≥25–30 | 1.000 | Reference | - |
| ≥30–35 | 1.261 | 1.217–1.305 | <0.0001 |
| ≥35–40 | 1.686 | 1.610–1.765 | <0.0001 |
| ≥40 | 2.371 | 2.247–2.501 | <0.0001 |
| Kt/V |  |  |  |
| <1.0 | 1.869 | 1.779–1.962 | <0.0001 |
| ≥1.0–1.2 | 1.286 | 1.239–1.336 | <0.0001 |
| ≥1.2–1.4 | 1.094 | 1.059–1.130 | <0.0001 |
| ≥1.4–1.6 | 1.000 | Reference | - |
| ≥1.6–1.8 | 0.968 | 0.933–1.004 | 0.081 |
| ≥1.8 | 0.839 | 0.805–0.875 | <0.0001 |

BP, blood pressure; CI, confidence interval; CVD, cardiovascular disease; HR, hazard ratio.
