# Supplementary material for: Current dialyzer classification in Japan and mortality risk in patients undergoing hemodialysis
Source: Sci Rep. 2024 May 4;14:10272. doi: 10.1038/s41598-024-60831-y (PMC11069571; doi:10.1038/s41598-024-60831-y)
Supplement: Supplementary file 7 — Supplementary Table S5. [file 41598_2024_60831_MOESM7_ESM.docx]

Supplementary Table S5. Hazard ratios of all-cause mortality in 181,804 patients receiving hemodialysis according to the dialyzer groups via Cox proportional hazards regression analysis

| Group | Unadjusted | | |  | Model 1 | | |  | Model 2 | | |  | Model 3 | | |
| --- | --- | --- | --- | --- | --- | --- | --- | --- | --- | --- | --- | --- | --- | --- | --- |
|  | HR | 95% CI | P value |  | HR | 95% CI | P value |  | HR | 95% CI | P value |  | HR | 95% CI | P value |
| Ia | 1.00 | Reference | - |  | 1.00 | Reference | - |  | 1.00 | Reference | - |  | 1.00 | Reference | - |
| IIa | 0.61 | 0.59–0.62 | <0.0001 |  | 0.76 | 0.74–0.78 | <0.0001 |  | 0.81 | 0.78–0.83 | <0.0001 |  | 0.91 | 0.87–0.93 | <0.0001 |
| IIb | 0.48 | 0.44–0.53 | <0.0001 |  | 0.69 | 0.63–0.77 | <0.0001 |  | 0.76 | 0.69–0.86 | <0.0001 |  | 0.87 | 0.78–0.97 | 0.009 |
| S | 1.39 | 1.34–1.45 | <0.0001 |  | 1.25 | 1.20–1.30 | <0.0001 |  | 1.17 | 1.12–1.23 | <0.0001 |  | 0.95 | 0.89–0.99 | 0.013 |

Model 1 was adjusted for basic factors including age, sex, dialysis vintage, and presence or absence of diabetes mellitus and cardiovascular complications. Model 2 was adjusted for dialysis-related factors including Kt/V values, β_2_-microglobulin levels, and systolic and diastolic blood pressure levels in addition to basic factors. Model 3 was adjusted for basic, dialysis-related, and nutrition- and inflammation-related factors including body mass index and C-reactive protein, hemoglobin, calcium, phosphate, intact parathyroid hormone, and serum albumin levels. CI, confidence interval; HR, hazard ratio.
